# Uniformly convergent extended cubic B-spline collocation method for two parameters singularly perturbed time-delayed convection-diffusion problems

**DOI:** 10.1186/s13104-023-06457-1

**Published:** 2023-10-19

**Authors:** Naol Tufa Negero

**Affiliations:** Department of Mathematics, Wollega University, 395 Nekemte, Oromia Ethiopia

**Keywords:** Singular perturbations, Two parameter convection-diffusion problem, Time delay, Exponentially fitted method, Extended cubic B-spline, Boundary layer, 65M06, 65M12, 65L11

## Abstract

This work proposes a uniformly convergent numerical scheme to solve singularly perturbed parabolic problems of large time delay with two small parameters. The approach uses implicit Euler and the exponentially fitted extended cubic B-spline for time and space derivatives respectively. Extended cubic B-splines have advantages over classical B-splines. This is because for a given value of the free parameter $\lambda$ the solution obtained by the extended B-spline is better than the solution obtained by the classical B-spline. To confirm the correspondence of the numerical methods with the theoretical results, numerical examples are presented. The present numerical technique converges uniformly, leading to the current study of being more efficient.

## Introduction

Consider the two-parameter singularly perturbed one-dimensional parabolic time delay convection-diffusion initial-boundary value problem defined as

1 $$\begin{aligned} \left\{ \begin{aligned}&\left( \frac{\partial }{\partial t} +L_{\varepsilon ,\mu }\right) u(x,t) =H(x,t), (x,t)\in {D} \\&u(x,t)={\phi _{b}(x,t)}, \; (x,t)\in \Gamma _{b}={{[0,1]}\times {[-\tau ,0]}},\\&u(0,t)={\phi _{l}(t)}, \; {\Gamma _{l}}=\lbrace {(0,t):{{0}\le {t}\le {T}}}\rbrace ,\\&u(1,t)={\phi _{r}(t)}, \; {\Gamma _{r}}=\lbrace {(1,t):{{0}\le {t}\le {T}}}\rbrace . \end{aligned} \right. \end{aligned}$$

where $D = \Omega _{x}\times {(0, T]}$, $\Omega _{x}= (0,1)$, ${0 <\varepsilon \le 1}, 0 \le \mu \le 1$, $H(x,t)=c(x,t)u(x,t-\tau )+f(x,t)$ and ${\tau >0}$ represents the delay parameter and *a*(*x*, *t*), *b*(*x*, *t*), *c*(*x*, *t*), *f*(*x*, *t*), $\phi _{b}(x,t), \phi _{l}(t)$ and $\phi _{r}(t)$ are sufficiently smooth, bounded functions on $\overline{D} =\left[ 0,1\right] \times \left[ 0,T\right] ,$ that satisfy

$$\begin{aligned} {a(x,t)}\ge {\alpha }> {0}, {b(x,t)}\ge {\beta }> {0}, {c(x,t)}\ge {\vartheta }> {0}, \gamma =\min _{{\bar{D}}}\left( \frac{b}{a} \right) . \end{aligned}$$

The operator $L_{\varepsilon ,\mu }$ given as

$$\begin{aligned} L_{\varepsilon ,\mu }{u(x,t)}\equiv -\varepsilon u_{xx}-\mu a(x,t)u_{x}+b(x,t)u. \end{aligned}$$

The existence of function approximations has been the subject of extensive research [1–14]. Here, the existence and uniqueness of a solution of (1) can be established under the assumption that the data are Holder continuous and sufficient smoothness of initial-boundary data on $\Gamma =\Gamma _{b}\cup \Gamma _{l}\cup \Gamma _{r}$ and compatibility conditions at the corner points $(0,0),(1,0),(0,-\tau )$ and $(1,-\tau )$, and delay terms [15].

2 $$\begin{aligned} \left\{ \begin{aligned}&\phi _{b}{(0,0)}=\phi _{l}{(0)},\\&{\phi _{b}{(1,0)}={\phi _{r}{(0)}}}, \end{aligned} \right. \end{aligned}$$

so that a unique solution exists and is sufficiently smooth for the model problem (1). For $\varepsilon \rightarrow 0$ and $\mu =1,$ numerical methods available in [16, 17] for the problem given by Eqs.(1) whose solution exhibits an exponential boundary layer of width $O\left( \varepsilon \right)$ in the left boundary layer $\Gamma _{l}$. As the parameters $\varepsilon \rightarrow 0$ and $\mu \rightarrow 0$, the solution develops boundary layers at $x = 0$ and $x = 1$. The parameters and the ratio $\varepsilon /\mu ^{2}$ affect the boundary layer’s width. We look at Eq. (1) above with $\varepsilon /\mu ^{2} \rightarrow 0$ as $\mu \rightarrow 0$ and $\mu ^{2}/\varepsilon \rightarrow 0$ as $\varepsilon \rightarrow 0$. As a result, the uniformly convergent numerical treatment presented in this study is independent of the two parameters $\varepsilon$ and $\mu$.

Two-parameter time delayed singularly perturbed parabolic problems have not been studied as extensively as one-parameter problems. Such type of problems are widespread in many phenomena of real life problems (see, for example, [18–20]) described by boundary layer problems. For singularly perturbed one-parameter partial differential equations many works have been delivered numerically in recent years (see, for example, [16, 21–33]). Not much numerical investigations have been done on two-parameter time delayed singularly perturbed parabolic problems. The work on two-parameter time delayed singularly perturbed parabolic problems have been started by Govindarao *et* *al*. [34], where they considered an upwind difference scheme on the Shishkin type meshes. First-order in both space and time numerical method was established. Sumit *et* *al*. [35] extend the works, where they considered a hybrid scheme for space consisting of central difference, upwind and midpoint operators on layer adapted piecewise uniform Shishkin mesh. Almost second-order in space and first order in time numerical method was established. Negero [36–40] also considered the problem similar to Sumit *et* *al*. and proposed numerical methods based on fitted operator methods on a uniform mesh, which improved the rate of convergence. However, for the problem under study, there are no known fitted extended cubic B-spline numerical methods. Here, the paper focus on exponentially fitted extended cubic B-spline for spatial discretization and the implicit Euler method for time discretization on uniform meshes. This is the more accurate compared to existing methods for the problem addressed in this work.

The paper is arranged as follows. Section "Preliminaries" presents the bounds on the derivatives and exact solution of Eq. (1). The discrete scheme are discussed in Section "Discretization of the problem". Section "Convergence analysis" deals with convergence and stability of the proposed numerical scheme. Numerical results are given in Section "Numerical examples and results" to illustrate the theory. The paper concludes with a discussion of the results obtained.

**Notations:** In this paper, we denote a generic positive constant by *C*, independent of mesh parameters $\mu$ and $\varepsilon$. The supremum norm on a domain *D* is defined as

$$\begin{aligned} \Vert \hslash \Vert _{\bar{D}}=\sup _{\left( x,t\right) \in \bar{D}}|\hslash (x,t)|. \end{aligned}$$

## Preliminaries

### Lemma 1

(Continuous maximum principle) Let *z*(*x*, *t*) $\in C^{2}\left( D\right) \cap C^{0}\left( \bar{D}\right)$, and assume that $z(x,t)\ge 0$, $\forall (x,t)\in {\Gamma } =\Gamma _{l}\cup \Gamma _{b}\cup \Gamma _{r}$. Then $\left( \frac{\partial }{\partial t} +L_{\varepsilon ,\mu }\right) z(x,t) \ge 0$ in *D* implies that $z(x,t)\ge 0$, $\forall (x,t)\in \bar{D}$.

*Proof* Let $\left( \zeta ^{*},\nu ^{*}\right) \in D$ such that $z\left( \zeta ^{*},\nu ^{*}\right) ={\min _{(x,t)\in \bar{D}}z\left( x,t \right) }<0.$ Then $\left( \xi ^{*},\vartheta ^{*} \right) \notin \Gamma$. Since at the point $\left( \xi ^{*},\vartheta ^{*} \right)$ function $\pi$ attains minimum, then, we have $z_{x}=z_{t}=0$ at $\left( \zeta ^{*},\nu ^{*}\right)$ and $z_{xx}\left( \zeta ^{*},\nu ^{*} \right) \ge 0$ and thus,

$$\begin{aligned}&\left( \frac{\partial }{\partial t} +L_{\varepsilon ,\mu }\right) z(\zeta ^{*},\nu ^{*}) =\frac{\partial z\left( \zeta ^{*},\nu ^{*}\right) }{\partial t} -\varepsilon \frac{\partial ^{2} z\left( \zeta ^{*},\nu ^{*}\right) }{\partial x^{2}}\\&\quad -\mu {a(\zeta ^{*},\nu ^{*})}\frac{\partial z\left( \zeta ^{*},\nu ^{*}\right) }{\partial x}+ {b(x,t)}z{(\zeta ^{*},\nu ^{*})}<0, \end{aligned}$$

which is a contradiction. This implies $z(x,t)\ge 0$ $\forall$ $(x,t)\in \bar{D}$. $\square$

### Lemma 2

[35] Let *u*(*x*, *t*) be the solution of problems (1) and *i*, *j* are any non-negative integers satisfying $0 \le i +3j \le 4$. Then,

$$\begin{aligned} \left\| \dfrac{\partial ^{i+j}u}{\partial x^{i}\partial t^{j}}\right\| _{\bar{D}} \le C\left\{ \begin{aligned}&\frac{1}{\left( \sqrt{\varepsilon }\right) ^{i}}, \text {if}~ \frac{{\mu }^{2}}{\varepsilon }\rightarrow 0 ~ \text {as}~ \varepsilon \rightarrow 0,\\&\left( \frac{\mu }{\varepsilon }\right) ^{i}\left( \frac{{\mu }^{2}}{\varepsilon }\right) ^{j}, \text {if}~\frac{{\varepsilon }}{\mu ^{2}} \rightarrow 0 ~ \text {as}~ \mu \rightarrow 0,\\ \end{aligned} \right. \end{aligned}$$

where *C* a positive constant independent of the parameters $\varepsilon$ and $\mu$.

## Discretization of the problem

### The time semi-discretization

For the time domain [0, *T*] equidistant mesh discretization with uniform step size $\Delta t$ is used such that

$$\begin{aligned} {\varOmega }^{M}_{t}=\left\{ t_{m}=m\Delta t, m=0,1,...,M, \Delta t =T/M \right\} , \end{aligned}$$

where *M* is mesh elements used on the interval [0, *T*]. The mesh for $\left[ -\tau ,T\right]$ is defined as

$$\begin{aligned} {\varOmega }^{s}_{t}=\left\{ t_{m}=m\Delta t, m=0,1,...,s,t_{s}=\tau , \Delta t =\tau /s \right\} . \end{aligned}$$

where *s* mesh elements used on the interval $\left[ -\tau , 0\right]$. Here, semi-discretizing the given problems (1) by applying implicit Euler scheme written as

3 $$\begin{aligned} \left\{ \begin{aligned}&\frac{U^{m}\left( x\right) -U^{m-1}\left( x\right) }{\Delta t}-\varepsilon \left( U_{xx}\right) ^{m}\left( x\right) -\mu a^{m}\left( x\right) \left( U_{x}\right) ^{m}\left( x\right) +b^{m}\left( x\right) U^{m}\left( x\right) \\&\quad =H^{m}\left( x\right) ,\\&U^{m}\left( 0\right) =\phi _{l}\left( t_{m}\right) , 0\le m\le M, x\in \Omega _{x}, \\&U^{m}\left( 1\right) =\phi _{r}\left( t_{m}\right) , 0\le m\le M,x\in \Omega _{x}, \\&U^{m}\left( x\right) =\phi _{b}\left( x,t_{m}\right) , -s\le m\le -1, x\in \Omega _{x}, \end{aligned} \right. \end{aligned}$$

where $H^{m}\left( x\right) =-c^{m}(x){ {U}^{m-s}\left( x\right) }+f^{m}\left( x\right)$, $0\le m\le M,x\in \Omega _{x}$ and $U^{m}(x)$ is the approximate solution of $u(x,t_{m})$ at (*m*)*th* time level. The Eq. (3) can be rewritten as

4 $$\begin{aligned} \left\{ \begin{aligned}&\left( 1+\Delta t L_{\varepsilon ,\mu }^{\Delta t}\right) U^{m}(x)=H(x,t_{m}),\\&U^{m}(0)={\phi _{l}(t_{m})},\;m=0,...,M,\\&U^{m}(1)={\phi _{r}(t_{m})},\;m=0,...,M, \\&U^{m}(x)={\phi _{b}(x,t_{m})},\; x\in \left( 0,1\right) , -\left( s+1 \right) \le m \le -1, \end{aligned} \right. \end{aligned}$$

where

$$\begin{aligned}&L_{\varepsilon ,\mu }^{\Delta t}=-\varepsilon \left( U_{xx}\right) ^{m} \left( x\right) -\mu a^{m}\left( x\right) \left( U_{x}\right) ^{m}\left( x\right) + b^{m}\left( x \right) U^{m}\left( x\right) \\&H\left( x,t_{m}\right) = -\Delta t c^{m}(x){{U}^{m-s}\left( x\right) }+\Delta tf^{m}\left( x\right) + U^{m}\left( x\right) . \end{aligned}$$

#### Lemma 3

(Semi-discrete maximum principle) Assume that $\Pi ^{m+1}\left( x\right) \in C^{2,1}\left( \bar{D}\right)$ such that $\Pi ^{m+1}\left( 0\right) \ge 0$ and $\Pi ^{m+1}\left( 1\right) \ge 0$. Then, $\left( 1+\Delta t {\pounds }_{\varepsilon ,\mu }^{\Delta t}\right) \Pi ^{m+1}\left( x\right) \ge 0$, $\forall x\in D$, implies that $\Pi ^{m+1}(x)\ge 0$, $\forall x\in \bar{D}$.

*Proof*

Assume $y^{*} \in {\bar{D}}$ such that $\Pi ^{m+1}\left( y^{*} \right) ={\min _{(x)\in {\bar{D}}}\Pi ^{m+1}\left( x\right) }$ and suppose $\Pi ^{m+1}\left( y^{*} \right) <0$. Now, it is clear that $y^{*}\notin \left\{ 0,1\right\}$, which implies that $y^{*} \in \left( 0,1\right)$. Therefore, we have $\frac{d}{dx}\left( \Pi ^{m+1}\left( y^{*}\right) \right) =0$ and $\frac{d^{2}}{dx^{2}}\left( \Pi \left( y^{*}\right) \right) \ge 0$ and thus

$$\begin{aligned}&\left( 1+\Delta t {\pounds }_{\varepsilon ,\mu }^{\Delta t}\right) \Pi ^{m+1}\left( y^{*} \right) =-{\varepsilon }\Delta t \frac{d^{2}}{dx^{2}}\left( \Pi ^{m+1}\left( y^{*}\right) \right) \\&\quad -\mu \Delta t{a^{m+1}\left( y^{*}\right) }\frac{d}{dx} \Pi ^{m+1}\left( y^{*}\right) +\left( 1+\Delta t \right) \Pi ^{m+1}\left( y^{*}\right) <0, \end{aligned}$$

this contradicts assumption and $\Pi ^{m+1}(y^{*})\ge 0$, which implies that $\Pi ^{m+1}(x)\ge 0,$ $\forall (x)\in \bar{D}$. $\square$

Let $u(x,t_{m})$ be the exact and $U^{m}(x)$ be the approximate solution of the problem in (1). The error estimates for the temporal semi-discretization (4) $E_{m+1}=U^{m}(x)-u\left( x,t_{m}\right)$ satisfy the following Lemma.

#### Lemma 4

(Local error estimate) The local error estimate with the semi-discretized problem (4) is given by

$$\begin{aligned} \left\| E_{m+1}\right\| _{\infty } \le C\left( \Delta t \right) ^{2}. \end{aligned}$$

*Proof*

Applying Taylor’s series expansion to $u\left( x,t_{m}\right)$ gives,

5 $$\begin{aligned} u(x,t_{m+1})=u(x,t_{m})+\Delta t u_{t}\left( x,t_{m} \right) +O\left( \left( \Delta t \right) ^{2} \right) . \end{aligned}$$

Substituting (5) into the continuous problems (1) gives,

$$\begin{aligned} \frac{u(x,t_{m+1})-u(x,t_{m})}{\Delta t}&=u_{t}\left( x,t_{m} \right) +O\left( \left( \Delta t \right) ^{2} \right) \\&= \varepsilon u_{xx}\left( x,t_{m+1} \right) +\mu a(x,t_{m+1})u_{x}(x,t_{m+1})\\&-b(x,t_{m+1})u\left( x,t_{m+1}\right) -c(x,t_{m+1})u\left( x,t_{-s+m}\right) +f(x,t_{m+1})\\&+O\left( \left( \Delta t \right) ^{2}\right) . \end{aligned}$$

Clearly $E_{m+1}(x)$ satisfies the semi-discrete operator

$$\begin{aligned} \left( 1+ \Delta t{L}_{\varepsilon ,\mu }^{\Delta t}\right) {E}_{m+1}(x)=O\left( \left( \Delta t \right) ^{2}\right) , \end{aligned}$$

with the conditions:

$$\begin{aligned} E_{m+1}(0)=E_{m+1}(1)=0. \end{aligned}$$

Thus using maximum principle given at Lemma 3 we have

$$\begin{aligned} \left\| E_{m+1}\right\| _{\infty } \le C\left( \Delta t \right) ^{2}. \end{aligned}$$

$$\square $$

#### Lemma 5

(Global error estimate.) The global error estimate $TE_{m}$ in the temporal direction at $t_{m}$ is given by

$$\begin{aligned} \left\| TE_{m}\right\| \le C\left( \Delta t \right) . \end{aligned}$$

*Proof*

The global error estimate at the $\left( m \right) th$ time step is given by

$$\begin{aligned} \left\| TE_{m}\right\| _{\infty }&=\left\| \sum _{k=1}^{m}{e_{k}}\right\| _{\infty }, m\le \dfrac{T}{\Delta t} \\&\le \left\| e_{1}\right\| _{\infty } +\left\| e_{2}\right\| _{\infty } +...+\left\| e_{m}\right\| _{\infty }. \end{aligned}$$

Using local error estimates given in Lemma 4,

$$\begin{aligned}&\le C_{1}\left( (m) \Delta t \right) \left( \Delta t \right) \\&\le C_{1} T\left( \Delta t \right) , \text {since }~m\left( \Delta t \right) \le T\\&\le C \left( \Delta t \right) ,C=C_{1} T, \end{aligned}$$

where *C* is constant independent of $\varepsilon$, $\mu$ and $\Delta t$. $\square$

#### Lemma 6

[41] The solution $U^{m}(x)$ of semi-discretized scheme (4) and its derivatives satisfies

$$\begin{aligned} \vert { \frac{d^{i}U^{m}(x)}{dx^{i}}}\vert \le C\left( 1+\omega _{1}^{-i}e^{-\nu \omega _{1} x }+\omega _{2}^{-i}e^{-\nu \omega _{2} \left( 1-x \right) } \right) , \text {for}~0\le i\le 4, \end{aligned}$$

where $\nu$ is any real constant number, $\lambda _{1}\left( x\right)$ and $\lambda _{2}\left( x\right)$ are two real solutions of (4) such that $\lambda _{1}\left( x\right) <0$ and $\lambda _{2}\left( x\right) >0$ and by assumption $\omega _{1}=-\max _{x\in \left[ 0,1 \right] }\lambda _{1}\left( x\right)$ and $\omega _{2}=\min _{x\in \left[ 0,1 \right] }\lambda _{2}\left( x\right)$.

### Discrete extended cubic B-splines construction

The spatial domain $\left[ 0,1\right]$ is discretized into *N* equal number of mesh elements each of length $h=N^{-1}$. This gives the spatial mesh

$$\begin{aligned} \Omega _{x}^{N}=\left\{ x_{n}=nh, n=1, 2, . . ., N, {x}_{0}=0,{x}_{N}=1\right\} , \end{aligned}$$

where $x_{n}$ is mesh points. The extended cubic B-spline basis of degree 4, $K_{n}\left( x,\lambda \right) ,$ is defined as the form

6 $$\begin{aligned} K_{n}\left( x ,\lambda \right) = \frac{1}{24h^{4}} \left\{ \begin{aligned}&4h\left( 1-\lambda \right) \left( x-x_{n-2} \right) ^{3}+3\lambda \left( x-x_{n-2} \right) ^{4}, x\in \left[ x_{n-2},x_{n-1} \right] ,\\&\left( 4-\lambda \right) h^{4}+12h^{3}\left( x-x_{n-1} \right) \\&+6h^{2}\left( 2+\lambda \right) \left( x-x_{n-1} \right) ^{2} -12h\left( x-x_{n-1} \right) ^{3} \\ {}&-3\lambda \left( x-x_{n-1} \right) ^{4}, x\in \left[ x_{n-1},x_{n} \right] ,\\&\left( 4-\lambda \right) h^{4}+12h^{3}\left( x_{n_{n+1}}-x \right) \\&+ 6h^{2}\left( 2+\lambda \right) \left( x_{n_{n+1}}-x \right) ^{2} -12h\left( x_{n_{n+1}}-x \right) ^{3} \\&-3\lambda \left( x_{n_{n+1}}-x \right) ^{4}, x\in \left[ x_{n},x_{n+1} \right] ,\\&4h\left( 1-\lambda \right) \left( x_{n+2}-x \right) ^{3}+3\lambda \left( x_{n+2}-x\right) ^{4}, x\in \left[ x_{n+1},x_{n+2} \right] ,\\&0, ~~~~\text {otherwise}. \end{aligned} \right. \end{aligned}$$

**Table 1:** Values of $K_{n}\left( x \right)$ and its first two derivatives at the nodal points

*x*	$x_{n-1}$	$x_{n}$	$x_{n+1}$	Otherwise
$K_{n}\left( x,\lambda \right)$	$\frac{4-\lambda }{24}$	$\frac{8+\lambda }{12}$	$\frac{4-\lambda }{24}$	0
$K_{n}^{'}\left( x,\lambda \right)$	$\frac{-1}{2h}$	0	$\frac{1}{2h}$	0
$K_{n}^{''}\left( x,\lambda \right)$	$\frac{2+\lambda }{2h^{2}}$	$-\frac{2+\lambda }{h^{2}}$	$\frac{2+\lambda }{2h^{2}}$	0

An approximation extended cubic B-spline function, $S(x,\lambda )$ to the exact solution $U\left( x,t_{m+1} \right)$ at $(m+1)$th time level is a linear combination of the extended cubic B-spline basis as

7 $$\begin{aligned} S(x,\lambda )=\sum _{n=-1}^{N+1}\zeta _{n}K_{n}\left( x,\lambda \right) , \end{aligned}$$

where $\zeta _{n}$’s are coefficients to be determined by collocation at each time level. Using the approximation given by (7) and Table 1 at nodal points $x=x_{m}$ in (4) gives, The Eq. (3) can be rewritten as

8 $$\begin{aligned} \left\{ \begin{aligned}&\left( 1+\Delta t L_{\varepsilon ,\mu }^{\Delta t,h}\right) U^{m+1}(x_{n})=H\left( x_{n},t_{m}\right) ,\\&U^{m+1}(0)={\phi _{l}(t_{m+1})},\;m=0,...,M,\\&U^{m+1}(1)={\phi _{r}(t_{m+1})},\;m=0,...,M, \\&U^{m+1}(x_{n})={\phi _{b}(x_{n},t_{m+1})},\; x_{n}\in \left( 0,1\right) , -\left( s+1 \right) \le m \le -1, \end{aligned} \right. \end{aligned}$$

where

$$\begin{aligned} L_{\varepsilon ,\mu }^{\Delta t,h}&=-\sigma \left( \varepsilon ,\mu \right) \left( U_{xx}\right) ^{m+1} \left( x_{n}\right) -\mu a^{m+1}\left( x_{n}\right) \left( U_{x}\right) ^{m+1}\left( x_{n}\right) \\&+ b^{m+1}\left( x_{n}\right) U^{m+1}\left( x_{n}\right) ,\\ H\left( x_{n},t_{m}\right)&= -\Delta t c^{m+1}(x_{n}){{U}^{m+1-s}\left( x_{n}\right) }+\Delta tf^{m+1}\left( x_{n}\right) + U^{m}\left( x_{n}\right) . \end{aligned}$$

Putting the approximation (7) into collocation (8) the operator $1+\Delta t L_{\varepsilon ,\mu }^{\Delta t,h}$ in (8) is given as

9 $$\begin{aligned} r_{n}^{-}\zeta _{n-1}+r_{n}^{c}\zeta _{n}+r_{n}^{+}\zeta _{n+1}=H\left( x_{n},t_{m}\right) , 0\le n\le N, \end{aligned}$$

where

$$\begin{aligned} \left\{ \begin{aligned} r_{n}^{-}&=-\sigma \left( \varepsilon ,\mu \right) \Delta t\frac{2+\lambda }{2h^{2}}+\mu \Delta t\frac{1}{2h}a^{m+1}\left( x_{n} \right) +\frac{4-\lambda }{24}\left( 1+ \Delta tb^{m+1}\left( x_{n}\right) \right) , \\ r_{n}^{c}&=\sigma \left( \varepsilon ,\mu \right) \Delta t\frac{2+\lambda }{h^{2}}+\frac{8+\lambda }{12}\Delta tb^{m+1}\left( x_{n} \right) , \\ r_{n}^{+}&=-\sigma \left( \varepsilon ,\mu \right) \Delta t\frac{2+\lambda }{2h^{2}}-\mu \Delta t\frac{1}{2h}a^{m+1}\left( x_{n} \right) +\frac{4-\lambda }{24}\left( 1+ \Delta tb^{m+1}\left( x_{n}\right) \right) , \\ \end{aligned} \right. \end{aligned}$$

where $$\sigma \left( \varepsilon ,\mu \right) =\varepsilon \frac{\rho \mu a_{m}}{2+\lambda }\coth {\left( \mu \frac{\rho a_{m}}{2}\right) }$$.

For the given boundary conditions we have

10 $$\begin{aligned} \left\{ \begin{aligned}&\frac{4-\lambda }{24}\zeta _{-1}+\frac{8+\lambda }{12}\zeta _{0}+\frac{4-\lambda }{24}\zeta _{1}=\phi _{l}\left( t_{m+1}\right) ,\\&\frac{4-\lambda }{24}\zeta _{N-1}+\frac{8+\lambda }{12}\zeta _{N}+\frac{4-\lambda }{24}\zeta _{N+1}=\phi _{r}\left( t_{m+1}\right) . \end{aligned} \right. \end{aligned}$$

The Eqs.(9, 10) gives to $\left( N+3\right) \times \left( N+3\right)$ systems in $\left( N+3\right)$ unknowns $\zeta _{-1},\zeta _{0},\zeta _{1},...,\zeta _{N+1}$. From Eqs. (9, 10), eliminating $\zeta _{-1}$ and $\zeta _{N+1}$ results $\left( N+1\right)$ system of equations in $\left( N+1\right)$ unknowns $\zeta _{0}, \zeta _{1},...,\zeta _{N}$ which can be written in a matrix form as

11 $$\begin{aligned} RV=Q, \end{aligned}$$

where

$$\begin{aligned} R=\left( \begin{array}{ccccccccc} -2\left( \frac{8+\lambda }{4-\lambda } \right) r_{0}^{-}+r_{0}^{c} &{}-r_{0}^{-}+r_{0}^{+} &{} 0 &{}\dots &{} \dots &{} \dots &{}\dots &{} 0\\ R_{1}\left( x_{1} \right) &{} R_{2}\left( x_{1} \right) &{}R_{3}\left( x_{1} \right) &{} 0 &{}0 &{} \dots &{} \dots &{}0\\ 0&{}R_{1}\left( x_{2} \right) &{} R_{2}\left( x_{2} \right) &{}R_{3}\left( x_{3} \right) &{} 0 &{} \dots &{} \dots &{} 0\\ \vdots &{}\ddots &{}\ddots &{} \ddots &{} \vdots &{}\vdots &{} \vdots &{} \vdots \\ 0&{}\dots &{}\dots &{}\dots &{}0&{} R_{1}\left( x_{N-1} \right) &{} R_{2}\left( x_{N-1} \right) &{}R_{3}\left( x_{N-1} \right) \\ 0&{} \dots &{} \dots &{} \dots &{} \dots &{} 0 &{} r_{N}^{-}-r_{N}^{+}&{}r_{N}^{c} -2\left( \frac{8+\lambda }{4-\lambda } \right) r_{N}^{+} \end{array}\right) , \end{aligned}$$

where $R_{n}\left( x_{n} \right) ,n=1,2,...,N-1$ are defined as

$$\begin{aligned} \left\{ \begin{aligned} R_{1}\left( x_{n} \right)&=-\sigma \left( \varepsilon ,\mu \right) \Delta t\frac{2+\lambda }{2h^{2}}+\mu \Delta t\frac{1}{2h}a^{m+1}\left( x_{n} \right) +\frac{4-\lambda }{24}\left( 1+ \Delta tb^{m+1}\left( x_{n}\right) \right) , \\ R_{2}\left( x_{n} \right)&=\sigma \left( \varepsilon ,\mu \right) \Delta t\frac{2+\lambda }{h^{2}}+\frac{8+\lambda }{12}\Delta tb^{m+1}\left( x_{n} \right) , \\ R_{3}\left( x_{n} \right)&=-\sigma \left( \varepsilon ,\mu \right) \Delta t\frac{2+\lambda }{2h^{2}}-\mu \Delta t\frac{1}{2h}a^{m+1}\left( x_{n} \right) +\frac{4-\lambda }{24}\left( 1+ \Delta tb^{m+1}\left( x_{n}\right) \right) , \end{aligned} \right. \end{aligned}$$

and column vectors *V* and *Q* are given as $V=\left[ \zeta _{0}, \zeta _{1},..., \zeta _{N}\right] ^{T}$ and

$$\begin{aligned}{} & {} Q=\Big [ H\left( x_{0},t_{m} \right) -\phi _{l}\left( t_{m+1} \right) r_{0}^{-}, H\left( x_{1},t_{m} \right) ,H\left( x_{2},t_{m} \right) ,..., \\{} & {} H\left( x_{N},t_{m} \right) -\phi _{r}\left( t_{m+1} \right) r_{N}^{+}\Big ] ^{T}. \end{aligned}$$

The matrix associated with Eq. (11) is of size $(N + 1)\times (N + 1)$ with its entries for $n = 1, 2,..., N-1$ are $R_{1}\left( x_{n} \right)<0, R_{2}\left( x_{n} \right) >0, R_{3}\left( x_{n} \right) <0.$ Therefore, the matrix *R* in Eq. (11) is an M-matrix and therefore its inverse exist and positive. Hence, tridiagonal system in Eq. (11) easily solved by any existing methods.

## Convergence analysis

### Lemma 7

The extended cubic B-splines $K_{-1}\left( x,\lambda \right)$, $K_{0}\left( x,\lambda \right)$, . . . $K_{N}\left( x,\lambda \right)$, $K_{N+1}\left( x,\lambda \right)$ satisfy $\sum _{n=-1}^{N+1} \vert { K_{n}\left( x,\lambda \right) }\vert \le 1.75, 0<x<1.$

*Proof*

At $$ x_{n} $$,

$$\begin{aligned}&\sum _{n=-1}^{N+1} \vert { K_{n}\left( x,\lambda \right) }\vert =\vert { K_{n-1}\left( x_{n},\lambda \right) }\vert +\vert { K_{n}\left( x_{n},\lambda \right) }\vert +\vert {K_{n+1}\left( x_{n},\lambda \right) }\vert \\&\quad =\frac{4-\lambda }{24}+\frac{8+\lambda }{12}+\frac{4-\lambda }{24}=1. \end{aligned}$$

For $$ x_{n-1}<x< x_{n+1} $$,

$$\begin{aligned}&\vert { K_{n}\left( x,\lambda \right) }\vert<\frac{8+\lambda }{12}, \vert { K_{n-1}\left( x,\lambda \right) }\vert<\frac{4-\lambda }{24},\\&\vert { K_{n+1}\left( x,\lambda \right) }\vert<\frac{4-\lambda }{24} , \\&\vert { K_{n-2}\left( x,\lambda \right) }\vert <\frac{4-\lambda }{24}. \end{aligned}$$

Thus, for $$ x_{n-1}<x< x_{n+1}$$,

$$\begin{aligned}&\sum _{n=-1}^{N+1} \vert { K_{n}\left( x,\lambda \right) }\vert =\vert { K_{n-1}\left( x_{n},\lambda \right) }\vert +\vert { K_{n}\left( x_{n},\lambda \right) }\vert \\&\quad +\vert { K_{n+1}\left( x_{n},\lambda \right) }\vert +\vert { K_{n-2}\left( x_{n},\lambda \right) }\vert =\frac{20+\lambda }{12}. \end{aligned}$$

Since $-8<\lambda <1$, so $\frac{20+\lambda }{12}\le 1.75$ and this complete the proof. $\square$

### Theorem 1

Let $u\left( x_{n},t_{m+1} \right)$ be the continuous solution of Eqs. (1) and (2) and $S\left( x,\lambda \right)$ be the collocation approximation from the space of splines to the solution $U^{m+1}\left( x \right)$ be the approximate solution of Eq. (3). Then, for sufficiently large *N*, the following error bound holds

$$\begin{aligned} \vert {{L}_{\varepsilon ,\mu }^{\Delta t,h}\left( {U}^{m+1}\left( x_{n} \right) -S\left( x_{n},\lambda \right) \right) }\vert \le CN^{-2}. \end{aligned}$$

*Proof*

Let $Z_{N}\left( x_{n} \right)$ be a unique spline interpolate to the solution $U^{m+1}\left( x_{n} \right)$ of the problem (3) given by

12 $$\begin{aligned} Z_{N}\left( x_{n} \right) =\sum _{n=-1}^{N+1}\bar{\zeta }_{n}K_{n}\left( x,\lambda \right) . \end{aligned}$$

The estimates given in [42] yields

13 $$\begin{aligned} \begin{aligned}\left\| U^{m+1}\left( x_{n} \right) -Z_{N}\left( x_{n} \right) \right\| _{\infty } \le C_{0}\left\| \frac{d^{4}U^{m+1}\left( x_{n} \right) }{dx^{4}}\right\| _{\infty }N^{-4} \\\left\| \frac{dU^{m+1}\left( x_{n} \right) }{dx}-\frac{dZ_{N}\left( x_{n} \right) }{dx}\right\| _{\infty } \le C_{1}\left\| \frac{d^{4}U^{m+1}\left( x_{n} \right) }{dx^{4}}\right\| _{\infty }N^{-3}\\\left\| \frac{d^{2}U^{m+1}\left( x_{n} \right) }{dx^{2}}-\frac{d^{2}Z_{N}\left( x_{n} \right) }{dx^{2}}\right\| _{\infty } \le C_{0}\left\| \frac{d^{4}U^{m+1}\left( x_{n} \right) }{dx^{4}}\right\| _{\infty }N^{-2}. \end{aligned} \end{aligned}$$

Using triangle inequality,

14 $$\begin{aligned} \begin{aligned}\left\| {U}^{m+1}\left( x_{n} \right) -S\left( x_{n},\lambda \right) \right\| _{\infty } \le \left\| U^{m+1}\left( x_{n} \right) -Z_{N}\left( x_{n} \right) \right\| _{\infty }\\+\left\| Z_{N}\left( x_{n} \right) -S\left( x_{n},\lambda \right) \right\| _{\infty }. \end{aligned} \end{aligned}$$

The collocating conditions are ${L}_{\varepsilon ,\mu }^{\Delta t,h}{U}^{m+1}\left( x_{n} \right) ={L}_{\varepsilon ,\mu }^{\Delta t,h}S\left( x_{n},\lambda \right) =H\left( x_{n},t_{m} \right)$. Assume that ${L}_{\varepsilon ,\mu }^{\Delta t,h}Z_{N}\left( x_{n}\right) =\bar{H}\left( x_{n},t_{m} \right)$ which satisfies the boundary conditions $Z_{N}\left( x_{1}\right) =Z_{N}\left( x_{N+1}\right)$. Then,

15 $$\begin{aligned} \begin{aligned}\vert { {L}_{\varepsilon ,\mu }^{\Delta t,h}{U}^{m+1}\left( x_{n} \right) - {L}_{\varepsilon ,\mu }^{\Delta t,h}Z_{N}\left( x_{n}\right) }\vert =\vert { {L}_{\varepsilon ,\mu }^{\Delta t,h}S\left( x_{n},\lambda \right) - {L}_{\varepsilon ,\mu }^{\Delta t,h}Z_{N}\left( x_{n}\right) }\vert \\\quad =\vert {-\varepsilon \left( \frac{d^{2}U^{m+1}\left( x_{n} \right) }{dx^{2}}-\sigma \left( \varepsilon ,\mu \right) \frac{d^{2}Z_{N}\left( x_{n} \right) }{dx^{2}} \right) }\vert \\\quad +\vert {-\mu a\left( x\right) \left( \frac{dU^{m+1}\left( x_{n} \right) }{dx}-\frac{dZ_{N}\left( x_{n} \right) }{dx} \right) +b^{m+1}\left( x\right) \left( U^{m+1}\left( x_{n} \right) - Z_{N}\left( x_{n} \right) \right) }\vert \\\quad \le \vert {\varepsilon }\vert \vert {\sigma \left( \varepsilon ,\mu \right) }\vert \left\| \frac{d^{2}U^{m+1}\left( x_{n} \right) }{dx^{2}} \right\| _{\infty } + \vert {\varepsilon }\vert \vert {\sigma \left( \varepsilon ,\mu \right) }\vert \left\| \frac{d^{2}U^{m+1}\left( x_{n} \right) }{dx^{2}}-\frac{d^{2}Z_{N}\left( x_{n} \right) }{dx^{2}}\right\| _{\infty }\\\quad + \vert {\mu }\vert \left\| a(x)\right\| _{\infty } \left\| \frac{dU^{m+1}\left( x_{n} \right) }{dx}-\frac{dZ_{N}\left( x_{n} \right) }{dx} \right\| _{\infty } +\left\| b^{m+1}\left( x\right) \right\| _{\infty }\left\| U^{m+1}\left( x_{n} \right) - Z_{N}\left( x_{n} \right) \right\| _{\infty }. \end{aligned} \end{aligned}$$

Using Lemma 1 and using Eq.(13)

$$\begin{aligned} \max _{x\in D}\vert { {L}_{\varepsilon ,\mu }^{\Delta t,h}{U}^{m+1}\left( x_{n} \right) - {L}_{\varepsilon ,\mu }^{\Delta t,h}Z_{N}\left( x_{n}\right) }\vert \le CN^{-2}, \end{aligned}$$

this is because $\vert {\sigma \left( \varepsilon ,\mu \right) -1 }\vert \le CN^{-2}.$ Equation (11) and ${L}_{\varepsilon ,\mu }^{\Delta t,h}{U}^{m+1}\left( x_{n} \right) - {L}_{\varepsilon ,\mu }^{\Delta t,h}Z_{N}\left( x_{n}\right)$ results

16 $$\begin{aligned} R\left( V-\bar{V}\right) = Q-\bar{Q}, \end{aligned}$$

where

$$\begin{aligned}&V-\bar{V}=\left( \varsigma _{0}-\bar{\varsigma }_{0},\varsigma _{1}-\bar{\varsigma }_{1},. . . ,\varsigma _{N}-\bar{\varsigma }_{N} \right) ,\\&Q-\bar{Q}=\Big (H\left( x_{0},t_{m} \right) - \bar{H}\left( x_{0},t_{m} \right) ,H\left( x_{1},t_{m} \right) - \bar{H}\left( x_{1},t_{m} \right) , . . . ,\\&H\left( x_{N},t_{m} \right) - \bar{H}\left( x_{N},t_{m} \right) \Big ). \end{aligned}$$

The matrices *R* is invertible, *i*.*e*,  $\vert {R^{-1}}\vert \le C$, and the boundary conditions are bounded. Therefore, Eqs. (15) and (16) results $\vert { V-\bar{V}}\vert \le CN^{-2}$. Thus, Eqs. (7) and (12) gives

$$\begin{aligned} \left\| S\left( x_{n},\lambda \right) -Z_{N}\left( x \right) \right\| _{\infty } =\vert {\varsigma _{n}-\bar{\varsigma }_{n}}\vert \sum _{n=0}^{N+2} \vert {K_{n}\left( x,\lambda \right) }\vert \le CN^{-2}. \end{aligned}$$

$$\square $$

### Theorem 2

Let $u\left( x_{n},t_{m+1}\right)$ be the solution of the continuous problem (1)-(2) and $U_{n}^{m+1}$ be the numerical solution of (8). Then, there exists a constant *C* such that the following uniform error estimate holds:

$$\begin{aligned} \sup _{0<\varepsilon \le 1}\max _{0\le n\le N,0\le m\le M}\vert { u\left( x_{n},t_{m+1}\right) -U_{n}^{m+1}}\vert \le C\left( \Delta t + N^{-2}\right) . \end{aligned}$$

*Proof*

The proof is the consequence of Lemma 5 and Theorem 1. $\square$

## Numerical examples and results

In this section, two numerical results are used to confirm the theoretical results using the proposed numerical scheme. The exact solution of the numerical example is not available. Therefore, double mesh principle is used to find the maximum absolute error $E_{\varepsilon ,\mu }^{N,M}$ and the corresponding convergence order $p^{N,M}_{\varepsilon ,\mu }$ as

$$\begin{aligned} E_{\varepsilon ,\mu }^{N,M}=\max _{0\le n\le N,0\le m\le M}\vert { U_{n}^{m+1}-U_{2n}^{2m+1}}\vert \text {and}~~~~p^{N,M}_{\varepsilon ,\mu }={\log _{2}}\left( \frac{E_{\varepsilon ,\mu }^{N,M}}{E_{\varepsilon ,\mu }^{2N,2M}}\right) . \end{aligned}$$

The uniform error before extrapolation $E^{N,M}$ and the corresponding uniform order of convergence before extrapolation $p^{N,M}$ by:

$$\begin{aligned} E^{N,M}=\max _{\varepsilon ,\mu }E^{N,M}_{\varepsilon ,\mu } ~~ \text {and}~~ p^{N,M}={log_{2}}\left( \frac{E^{N,M}}{E^{2N,2M}}\right) , \end{aligned}$$

where $U_{m}^{n+1}$ is a numerical solution obtained using the space and time $N \times M$ mesh spacing with a mesh size of *h* or $\Delta t$.

*Example 1*

Consider problem

$$\begin{aligned}&\frac{\partial u}{\partial t}-\varepsilon \frac{\partial ^2 u}{\partial x^2}-\mu (1+x)\frac{\partial u}{\partial x}+u(x,t) =u(x,t-\tau )\\&\quad -16x^{2}\left( 1-x \right) ^{2},(x,t)\in (0,1)\times (0,2], \end{aligned}$$

with

$$\begin{aligned} \left\{ \begin{aligned}&u(0,t)=0, u(1,t)=0,t\in \left( 0,2\right] ,\\&u(x,t)=0,(x,t)\in \left[ 0,1\right] \times \left[ -\tau ,0\right] . \end{aligned} \right. \end{aligned}$$

*Example 2*

Consider problem

$$\begin{aligned}&\frac{\partial u}{\partial t}-\varepsilon \frac{\partial ^2 u}{\partial x^2}-\mu \left( 1+x\left( 1-x \right) +t^{2} \right) \frac{\partial u}{\partial x}+\left( 1+5xt\right) u(x,t)\\&\quad =u(x,t-\tau )+x(1-x)\left( e^{t} -1 \right) ,\\&\quad (x,t)\in (0,1)\times (0,2], \end{aligned}$$

with

$$\begin{aligned} \left\{ \begin{aligned}&u(0,t)=0, u(1,t)=0,t\in \left( 0,2\right] ,\\&u(x,t)=0,(x,t)\in \left[ 0,1\right] \times \left[ -\tau ,0\right] . \end{aligned} \right. \end{aligned}$$

**Fig. 1:**
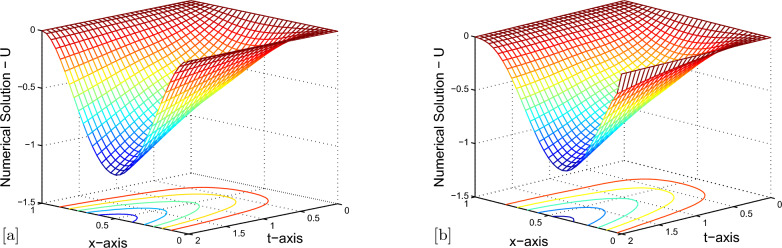
Surface plot of the numerical solution for Example 1 with $N=M=32$, (**a**) $\varepsilon = 10^{-1}$, $\mu = 10^{-6}$ (**b**) $\varepsilon = 10^{-6}$, $\mu = 10^{-1}.$

**Fig. 2:**
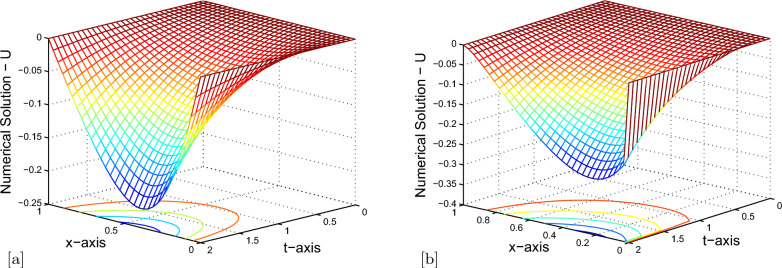
Surface plot of the numerical solution for Example 2 with $N=M=32$, (**a**) $\varepsilon = 10^{-1}$, $\mu = 10^{-6}$ (**b**) $\varepsilon = 10^{-6}$, $\mu = 10^{-1}.$

**Fig. 3:**
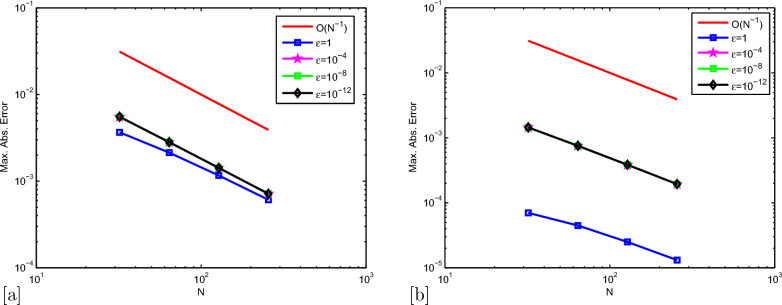
Log-Log plot of the maximum error on left (**a**) for Example 1 with $\mu = 10^{-4}$ and on right (**b**) for Example 2 with $\mu = 10^{-4}$

Maximum pointwise errors $(E^{N,M}_{\varepsilon ,\mu })$ and rate of convergence $(p^{N,M}_{\varepsilon ,\mu })$ for Example 1 and Example 2 have been demonstrated by fixing $\mu =10^{-4}$ and $\lambda =-1e-03$ in Tables 2, 3 respectively, for various values of $\varepsilon$. The results given in Tables 2, 3 clearly indicate that the proposed numerical method is accurate of order $O\left( \left( \Delta t\right) +N^{-2} \right)$. Also, tabulated results in Tables 4, 5 indicates that maximum point-wise errors going to stabilized as the two parameters $\mu$ and $\varepsilon$ approaches to zero. Comparisons of our numerical results with those of [35] are presented in Tables 6, 7. From these tables, we can confirm the more accurate of the proposed numerical method. The numerical solutions obtained by the numerical scheme presented in Example 1 are shown in Fig. 1a, b and numerical scheme presented in Example 2 are shown in Fig. 2a, b. From Figs. 1a,  2a, we confirm the occurrence of both left and right boundary layers near $x = 0$ and $x = 1$ for $\mu = 10^{-6}$ and boundary layers near $x = 0$ for $\mu = 10^{-1}$. The graphs between *N* and maximum pointwise errors of Examples 1 and 2 are plotted as the log-log scale respectively, in Fig. 3a, b. From these two graphs, one can observe that the numerical scheme converges uniformly as the perturbation parameters goes very small.

**Table 2:** $E_{\varepsilon ,\mu }^{N,M}$ and $p^{N,M}_{\varepsilon ,\mu }$ with $\mu =10^{-4}, \lambda =-1e-03,$ for Example 1

$$\varepsilon \downarrow $$	$$ N=32$$	$$ N=64 $$	$$ N=128 $$	$$ N=256 $$
	$\Delta t=0.25/2$	$\Delta t=0.25/2^{2}$	$\Delta t=0.25/2^{3}$	$\Delta t=0.25/2^{4}$
$10^{-0}$	$5.7908e-03$	$3.6490e-03$	$2.1309e-03$	$1.1633e-03$
	0.66626	0.77604	0.87324	
$10^{-2}$	$1.0523e-02$	$5.4742e-03$	$2.7938e-03$	$1.4116e-03$
	0.94283	0.97042	0.98490	
$10^{-4}$	$1.0658e-02$	$5.5311e-03$	$2.8188e-03$	$1.4230e-03$
	0.94630	0.97249	0.98615	
$10^{-6}$	$1.0662e-02$	$5.5324e-03$	$2.8191e-03$	$1.4231e-03$
	0.94650	0.97267	0.98620	
$10^{-8}$	$1.0663e-02$	$5.5336e-03$	$2.8200e-03$	$1.4237e-03$
	0.94632	0.97252	0.98605	
$10^{-10}$	$1.0663e-02$	$5.5336e-03$	$2.8200e-03$	$1.4237e-03$
	0.94632	0.97252	0.98605	
$10^{-12}$	$1.0663e-02$	$5.5336e-03$	$2.8200e-03$	$1.4237e-03$
	0.94632	0.97252	0.98605	
$E_{\varepsilon ,\mu }^{N,M}$	$1.0663e-02$	$5.5336e-03$	$2.8200e-03$	$1.4880e-03$
$p_{\varepsilon ,\mu }^{N,M}$	0.94632	0.97252	0.92232	-

**Table 3:** $E_{\varepsilon ,\mu }^{N,M}$ and $p^{N,M}_{\varepsilon ,\mu }$ with $\mu =10^{-4}, \lambda =-1e-03,$ for Example 2

$\varepsilon \downarrow$	$N=32$	$N=64$	$N=128$	$N=256$
	$\Delta t=0.25/2$	$\Delta t=0.25/2^{2}$	$\Delta t=0.25/2^{3}$	$\Delta t=0.25/2^{4}$
$10^{-0}$	$1.5539e-04$	$9.3152e-05$	$5.0828e-05$	$2.6527e-05$
	0.73824	0.87396	0.93816	
$10^{-2}$	$2.1188e-03$	$1.1511e-03$	$5.9904e-04$	$3.0557e-04$
	0.88023	0.94229	0.97115	
$10^{-4}$	$2.6785e-03$	$1.4493e-03$	$7.5362e-04$	$3.8430e-04$
	0.88607	0.94345	0.97160	
$10^{-6}$	$2.6752e-03$	$1.4491e-03$	$7.5411e-04$	$3.8487e-04$
	0.88449	0.94231	0.97040	
$10^{-8}$	$2.6752e-03$	$1.4489e-03$	$7.5355e-04$	$3.8426e-04$
	0.88469	0.94318	0.97162	
$10^{-10}$	$2.6752e-03$	$1.4489e-03$	$7.5355e-04$	$3.8426e-04$
	0.88469	0.94318	0.97162	
$10^{-12}$	$2.6752e-03$	$1.4489e-03$	$7.5355e-04$	$3.8426e-04$
	0.88469	0.94318	0.97162	
$E_{\varepsilon ,\mu }^{N,M}$	$2.6752e-03$	$1.4493e-03$	$5.9904e-04$	$3.8426e-04$
$p_{\varepsilon ,\mu }^{N,M}$	0.88429	1.2746	0.64057	-

**Table 4:** $E^{N,M}_{\varepsilon ,\mu }$ and $p^{N,M}_{\varepsilon ,\mu }$ with $\lambda =-1e-03,$ for Example 1

$\varepsilon \downarrow \mu \rightarrow$	$N=32$	$N=64$	$N=128$	$N=256$
	$\Delta t=0.125/2$	$\Delta t=0.125/2^{2}$	$\Delta t=0.125/2^{3}$	$\Delta t=0.125/2^{4}$
	$10^{-4}$	$10^{-6}$	$10^{-8}$	$10^{-10}$
$10^{-2}$	$5.5116e-03$	$2.8029e-03$	$1.4137e-03$	$7.1003e-04$
	0.97555	0.98744	0.99352	
$10^{-4}$	$5.5305e-03$	$2.8183e-03$	$1.4228e-03$	$7.1485e-04$
	0.97258	0.98609	0.99302	
$10^{-6}$	$5.5341e-03$	$2.8182e-03$	$1.4228e-03$	$7.1485e-04$
	0.97357	0.98604	0.99302	
$10^{-8}$	$5.5349e-03$	$2.8182e-03$	$1.4228e-03$	$7.1485e-04$
	0.97378	0.98604	0.99302	
$10^{-10}$	$5.5349e-03$	$2.8182e-03$	$1.4228e-03$	$7.1485e-04$
	0.97378	0.98604	0.99302	
$10^{-12}$	$5.5349e-03$	$2.8182e-03$	$1.4228e-03$	$7.1485e-04$
	0.97378	0.98604	0.99302	
$E_{\varepsilon ,\mu }^{N,M}$	$5.5349e-03$	$2.8183e-03$	$1.4228e-03$	$7.1485e-04$
$p_{\varepsilon ,\mu }^{N,M}$	0.97378	0.98604	0.99302	-

**Table 5:** $E^{N,M}_{\varepsilon ,\mu }$ and $p^{N,M}_{\varepsilon ,\mu }$ with $\lambda =-1e-03,$ for Example 2

$\varepsilon \downarrow \mu \rightarrow$	$N=32$	$N=64$	$N=128$	$N=256$
	$\Delta t=0.125/2$	$\Delta t=0.125/2^{2}$	$\Delta t=0.125/2^{3}$	$\Delta t=0.125/2^{4}$
	$10^{-4}$	$10^{-6}$	$10^{-8}$	$10^{-10}$
$10^{-0}$	$7.0128e-05$	$4.4746e-05$	$2.4968e-05$	$1.3154e-05$
	0.64823	0.84168	0.92458	
$10^{-2}$	$1.1458e-03$	$5.9706e-04$	$3.0501e-04$	$1.5414e-04$
	0.94041	0.96902	0.98462	
$10^{-4}$	$1.4510e-03$	$7.5362e-04$	$3.8417e-04$	$1.9399e-04$
	0.94514	0.97209	0.98576	
$10^{-6}$	$1.4434e-03$	$7.5568e-04$	$3.8530e-04$	$1.9454e-04$
	0.93362	0.97179	0.98592	
$10^{-8}$	$1.4434e-03$	$7.5566e-04$	$3.8531e-04$	$1.9454e-04$
	0.93366	0.97172	0.98595	
$10^{-10}$	$1.4434e-03$	$7.5566e-04$	$3.8531e-04$	$1.9454e-04$
	0.93366	0.97172	0.98595	
$10^{-12}$	$1.4434e-03$	$7.5566e-04$	$3.8531e-04$	$1.9454e-04$
	0.93366	0.97172	0.98595	
$E_{\varepsilon ,\mu }^{N,M}$	$1.1458e-03$	$7.5568e-04$	$3.8531e-04$	$1.9454e-04$
$p_{\varepsilon ,\mu }^{N,M}$	0.93366	0.97172	0.98595	-

**Table 6:** $E^{N,M}_{\varepsilon ,\mu }$ and $p^{N,M}_{\varepsilon ,\mu }$ with $\mu =10^{-3}, \lambda =0,$ for Example 1

$\varepsilon \downarrow$	$N=32$	$N=64$	$N=128$	$N=256$
	$\Delta t=0.25$	$\Delta t=0.25/2^{2}$	$\Delta t=0.25/2^{4}$	$\Delta t=0.25/2^{6}$
$10^{-0}$	$7.3970e-03$	$3.6490e-03$	$1.1642e-03$	$3.1262e-04$
	1.0194	1.6482	1.8969	
$10^{-2}$	$1.9438e-02$	$5.4750e-03$	$1.4141e-03$	$3.5651e-04$
	1.8279	1.9530	1.9879	
$10^{-4}$	$1.9861e-02$	$5.5330e-03$	$1.4235e-03$	$3.5849e-04$
	1.8438	1.9586		
$10^{-6}$	$1.9905e-02$	$5.5564e-03$	$1.4355e-03$	$3.6396e-04$
	1.8409	1.9526	1.9797	
$10^{-8}$	$1.9905e-02$	$5.5564e-03$	$1.4355e-03$	$3.6459e-04$
	1.8409	1.9526	1.9772	
$10^{-10}$	$1.9905e-02$	$5.5564e-03$	$1.4355e-03$	$3.6459e-04$
	1.8409	1.9526	1.9772	
$10^{-12}$	$1.9905e-02$	$5.5564e-03$	$1.4355e-03$	$3.6459e-04$
	1.8409	1.9526	1.9772	
$E_{\varepsilon ,\mu }^{N,M}$	$1.9905e-02$	$5.5564e-03$	$1.4355e-03$	$3.6459e-04$
Method in [35]				
$E_{\varepsilon ,\mu }^{N,M}$	$4.3706e-02$	$7.3807e-03$	$1.8967e-03$	$4.7927e-04$

**Table 7:** $E^{N,M}_{\varepsilon ,\mu }$ and $p^{N,M}_{\varepsilon ,\mu }$ with $\mu =10^{-9}, \lambda =0,$ for Example 2

$\varepsilon \downarrow$	$N=32$	$N=64$	$N=128$	$N=256$
	$\Delta t=0.25$	$\Delta t=0.25/2^{2}$	$\Delta t=0.25/2^{4}$	$\Delta t=0.25/2^{6}$
$10^{-0}$	$2.8379e-04$	$9.3145e-05$	$2.4962e-05$	$6.3499e-06$
	1.6073	1.8997	1.9749	
$10^{-2}$	$3.6086e-03$	$1.1504e-03$	$3.0489e-04$	$7.7337e-05$
	1.6493	1.9158	1.9791	
$10^{-4}$	$4.5529e-03$	$1.4480e-03$	$3.8396e-04$	$9.7429e-05$
	1.6527	1.9150	1.9785	
$10^{-6}$	$4.5651e-03$	$1.4523e-03$	$3.8509e-04$	$9.7695e-05$
	1.6523	1.9151	1.9788	
$10^{-8}$	$4.5652e-03$	$1.4523e-03$	$3.8510e-04$	$9.7698e-05$
	1.6523	1.9150	1.9788	
$10^{-10}$	$4.5652e-03$	$1.4523e-03$	$3.8510e-04$	$9.7698e-05$
	1.6523	1.9150	1.9788	
$10^{-12}$	$4.5652e-03$	$1.4523e-03$	$3.8510e-04$	$9.7698e-05$
	1.6523	1.9150	1.9788	
$E_{\varepsilon ,\mu }^{N,M}$	$4.5652e-03$	$1.4523e-03$	$3.8510e-04$	$9.7698e-05$
Method in [35]				
$E_{\varepsilon ,\mu }^{N,M}$	$1.1100e-02$	$2.4588e-03$	$6.0458e-04$	$1.5049e-04$

## Conclusion

In this paper, the exponentially fitted strategy is applied to extended cubic B-spline scheme for solving a two-parameter singularly perturbed temporal delay parabolic problem. In our present study of continuous problem, the temporal direction is discretized by an implicit-Euler scheme with a uniform mesh, and the spatial direction is discretized by an exponentially fitted extended cubic B-spline finite difference method fitting only one parameter $\varepsilon$. We have proved that the method provides first-order and second-order accurate uniformly convergent in time and space respectively. Two numerical tests are introduced to confirm the effectiveness of the proposed numerical scheme and approve the theoretical findings.

## Limitations

The proposed uniformly convergent numerical approach is based on a uniform mesh that does not resolve boundary layers because there are not a sufficient number of mesh points in boundary regions.

## Data Availability

No additional data is used for this research work.
